# Simultaneous Determination of Dopamine, Serotonin and Ascorbic Acid at a Glassy Carbon Electrode Modified with Carbon-Spheres

**DOI:** 10.3390/s131014029

**Published:** 2013-10-16

**Authors:** Jianqing Zhou, Meili Sheng, Xueyue Jiang, Guozhi Wu, Feng Gao

**Affiliations:** 1 Laboratory of Optical Probes and Bioelectrocatalysis (LOPAB), Key Laboratory of Chemo/Biosensing, Anhui Province, College of Chemistry and Materials Science, Anhui Normal University, Wuhu 241000, China; E-Mails: vineyardah@gmail.com (J.Z.); seemly@mail.ahnu.edu.cn (M.S.); jiangxueyue@126.com (X.J.); allen_0688@163.com (G.W.); 2 Department of Pharmacy, Anhui Medical College, Hefei 230601, China; 3 College of Chemistry and Chemical Engineering, Fuyang Teachers College, Fuyang 236041, China; 4 Department of Chemistry and Food Science, Chizhou College, Chizhou 247000, China

**Keywords:** carbon-spheres, simultaneous determination, dopamine, ascorbic acid, serotonin

## Abstract

A novel glassy carbon electrode (GCE) modified with carbon-spheres has been fabricated through a simple casting procedure. The modified GCE displays high selectivity and excellent electrochemical catalytic activities towards dopamine (DA), serotonin (5-HT), and ascorbic acid (AA). In the co-existence system, the peak separations between AA and DA, DA and 5-HT, and AA and 5-HT are large up to 230, 180, and 410 mV, respectively. Differential pulse voltammetry (DPV) has been employed to simultaneously detect DA, 5-HT, and AA, and the linear calibration curves for DA, 5-HT, and AA are obtained in the range of 20.0–150.0 μM, 40.0–750.0 μM and 300.0–2,000.0 μM with detection limits (S/N = 3) of 2.0 μM, 0.7 μM and 0.6 μM, respectively. The proposed electrode has been applied to detect DA, 5-HT, and AA in real samples using standard addition method with satisfactory results.

## 1. Introduction

Neurotransmitters, such as dopamine (3,4-dihydroxyphenylethylamine, DA) and serotonin (5-hydroxytryptamine, 5-HT) are the chemical messengers which transmit messages from one neuron to the next. This transmission proceeds through the secretion of neurotransmitters from one neuron and then binding to the specific receptor located on the membrane of the target cell. This interaction between neurotransmitter and receptor is one of the major modes of communication between neurons [1,2]. Dopamine is one of the most typical catecholamines and belongs to the family of inhibitory neurotransmitters. A loss of DA-containing neurons may result in some serious diseases such as schizophrenia and Parkinson's disease [3,4]. 5-HT is widely distributed in the brain, and together with other neurotransmitters, makes a significant contribution to brain functions such as sleep, thermoregulation, food intake, and sexual activity, as well as in psychopathological states such as depression, anxiety, alcoholism, and drug dependency [5,6]. Ascorbic acid (AA) has a significant role in normal neuronal physiology and acts as an important antioxidant, enzyme co-factor, and neuromodulator in the brain [7]. These three substances are all electro-active and coexist in biological systems such as brain tissue. There has been a considerable effort to develop voltammetric methods for the determination of AA, DA, and 5-HT in real biological matrixes. It is well known that the direct redox reactions of these three species at bare electrodes are irreversible and therefore require high overpotentials. Moreover, the direct redox reactions of these species at the bare electrodes take place at very similar potentials and they suffer from a pronounced fouling effect, which results in rather poor selectivity and reproducibility [8–12], so the ability to selectively detect DA, 5-HT and AA, has been a matter of great interest to bioelectrochemists, electroanalytical chemists and neuroscientists [8–12]. Up to now, there are a few reports in the literature about the simultaneous determination of DA, 5-HT and AA which used different electrochemical methods and electrodes for their simultaneous determination. For example, various chemically modified electrodes including electrodeposited nanostructured platinum on Nafion-coated GCEs [13], poly(*o*-phenylenediamine)- and poly(phenosafranine)-modified GCEs [14,15], choline- and acetylcholine-modified GCEs [16], DNA-immobilized carbon fibre microelectrodes [17,18], solid carbon paste electrode modified with a nonionic polymer film [19], and carbon paste electrodes modified with iron(II) phthalocyanine complexes [20] have been reported for the simultaneous determination of DA, 5-HT and AA. A proposed methodology for the discrimination between DA and 5-HT in the presence of AA with the combination of a large amplitude/ high frequency voltage excitation and signal processing techniques [21] is also reported in the literature. However, direct use of carbon materials (*i.e.*, without other modifiers) for the simultaneous determination of these three species have rarely been reported [22–26]. To the best our knowledge, only carbon-nanotube-modified GCEs [22], carbon-nanotube-intercalated graphite electrodes [23], graphite electrodes reinforced by carbon [24] edge plane pyrolytic graphite electrode [25], and carbon nanofiber [26] have been employed as simple electroanalytical methodology for the simultaneous determination of dopamine, serotonin and ascorbic acid.

Recently, we have synthesized a new kind of micro-structured porous carbon materials, double-shelled carbon spheres (CS), and we have shown their excellent electrochemical properties as electrode materials such as good conductivity, porous nature and high specific area [27–30]. Herein, the feasibility of using a CS-modified glassy carbon electrode(CS/GCE) in an attempt to develop a sensitive voltammetric method for the simultaneous determination of DA, 5-HT and AA in pH 7.00 phosphate buffer solution is reported.

## 2. Experimental Section

### 2.1. Chemicals and Instrumentation

Ascorbic acid (AA), dopamine (DA), serotonin (5-HT) and other chemicals were all purchased from Sinopharm Chemical Reagent Co., Ltd (Shanghai, China). All these chemicals are of analytical grade and used as received. Unless otherwise indicated, phosphate buffer (pH 7.0, 0.1 M) was used as supporting electrolyte. Phosphate buffer was prepared with KH_2_PO_4_ and Na_2_HPO_4_, and the desired pH was modulated with NaOH or H_3_PO_4_ by pH meter. Freshly prepared AA, DA and 5-HT solutions were used for each experiment. All aqueous solutions were prepared with triply-distilled water. Morphological characterization of the obtained carbon spheres was performed on a transmission electron microscope (SEM) (Hitachi S-4800, Tokyo, Japan) operating at 3 kv. A very dilute dispersion of the carbon spheres in ethanol was dispersed onto carbon-coated copper grids and the ambient dried carbon spheres were vacuum sputtered for SEM observation. All electrochemical experiments were performed on CHI760D electrochemical working station (CHI, Shanghai, China) with a conventional three-electrode cell which consists of a bare GCE or CS/GCE as the working electrode, a Ag/AgCl as the reference electrode, and a platnium wire as the counter electrode.

### 2.2. Preparation of CS Film Electrodes

Prior to surface modification, the glassy carbon disk electrodes (GC, 3-mm diameter, Bioanalytical System, Inc., West Lafayette, IN, USA) were first polished with 0.3 and 0.05 μm alumina slurry on a polishing cloth, respectively, and then sonicated in the acetone and distilled water for 3 min, respectively. The as-synthesized CS was dispersed into N,N-dimethylformamide (DMF) to give a homogeneous suspension (10 mg·mL^−1^) under sonication. A 2.0 μL aliquot of the homogeneous suspension was cast onto the GCE surface and allowed to dry under a lamp to evaporate the solvent, thus a CS-modified GCE (denoted as CS/GCE) was obtained.

## 3. Results and Discussion

### 3.1. Characterization of Synthesized Carbon Spheres

**Figure 1.**
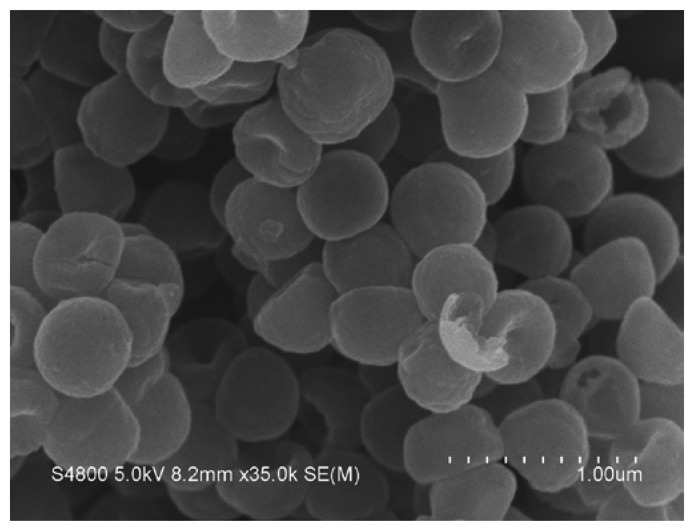
SEM image of the synthesized carbon spheres.

The synthesis of carbon spheres and the detailed characterization of the synthesized carbon spheres including SEM, TEM, IR spectra, elemental analysis, Raman spectra, and BET tests were reported in our previous publications [26–29]. In brief, the carbon spheres with porous shell are about 480 nm in diameter. BET specific surface area was 194 m^2^/g, and the total pore volume was 0.36 cm^3^/g. Elemental analysis shows that the carbon spheres contain 94.0 wt.% C, 1.0 wt.% H, 2.7 wt.% O and 1.0 wt.% S, suggesting that oxygen-containing functional groups are present on the CS surface. The SEM image of the synthesized carbon sphere is shown in Figure 1.

### 3.2. Electrochemical Responses of AA, DA and 5-HT on CS/GCE

**Figure 2.**
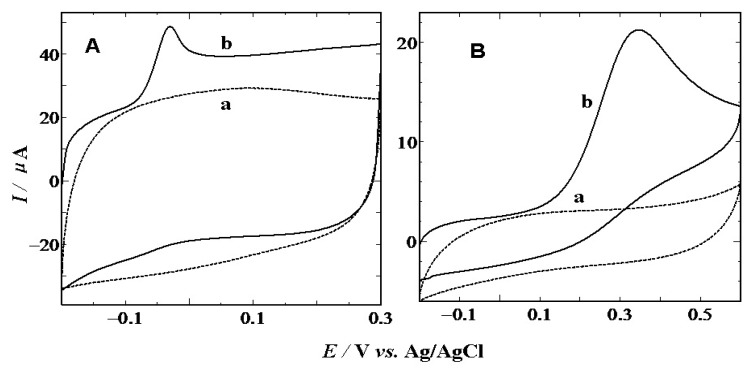
Cyclic voltammograms obtained at CS/GCE (**A**), and bare GCE (**B**) in the presence(solid line, curve b) or absence(dotted line, curve a) of 1 mM of AA in the phosphate buffer solution (pH 7.0) with a scan rate of 100 mV·s^−1^.

Figure 2 depicts cyclic voltammograms (CVs) of the electro-oxidation of 1 mM AA at the CS modified (A) and bare (B) GCEs in 0.1 mol·L^−1^ PBS (pH 7.0) with a scan rate of 100 mV·s^−1^, respectively. At a bare GCE, AA shows a broad and irreversible oxidation peak at 0.35 V, while the oxidation peak shifted to −0.03 V with well-defined peak shape at the CS/GCE. The 380 mV negative shift of the anodic peak indicates that the CS modified GCE also plays a strong catalytic effect on the AA electro-oxidation.

In the electrooxidation process of AA, ascorbate is oxidized to dehydroascorbate accompanied by with transfer of 2-electrons and 2-protons, and the electrochemical reaction can be expressed as follows:

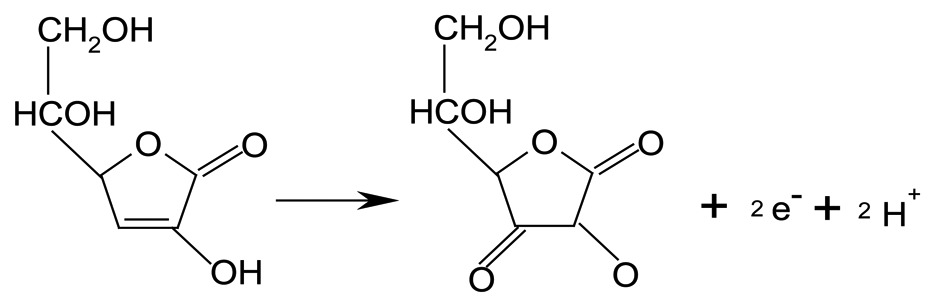


**Figure 3.**
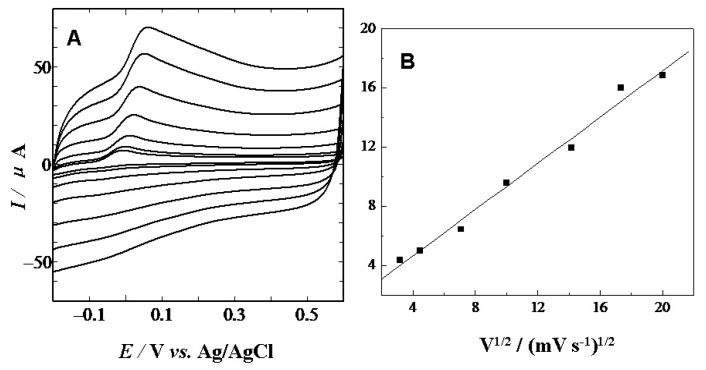
(**A**) CVs obtained at CS/GCE of 1 mM of AA in the phosphate buffer solution (pH 7.0) at different scan rates (from inner to outer): 10, 20, 50, 100, 200, 300, 400 mV·s^−1^; (**B**) The plot of currents against the square roots of scan rates.

As shown in Figure 3, the anodic peak currents were proportional to the square roots of scan rates in the range of 10–400 m·Vs^−1^, indicating that the electrochemical reactions of AA at CS/GCE is a diffusion-controlled process.

**Figure 4.**
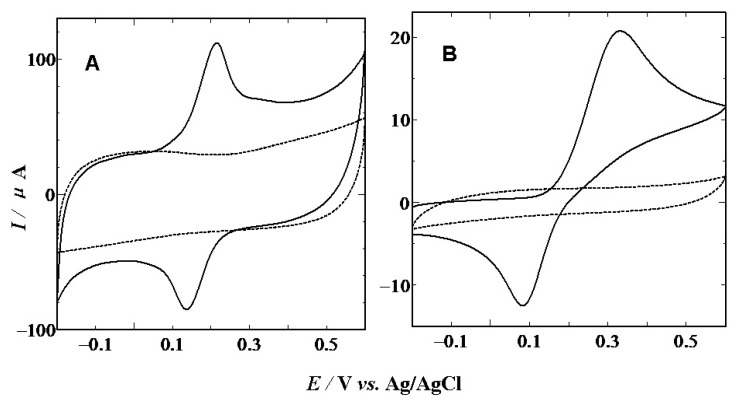
CVs obtained at CS/GCE (**A**), and bare GCE (**B**) in the presence (solid line) or absence (dotted line) of 1 mM of DA in the phosphate buffer solution (pH 7.0) with a scan rate of 100 m·Vs^−1^.

Figure 4 shows the cyclic voltammmograms of 1 mM DA at CS modified (A) and bare (B) GCEs in the phosphate buffer solution (pH 7.0) with a scan rate of 100 mV·s^−1^, respectively. As can be seen, DA shows a sluggish and much smaller CV peak response with a ΔE_p_ of 0.35 V at bare GCE (B). But at the CS modified GCE, the peak potential shifted negatively and showed a pair of quasi-reversible redox peaks with a ΔE_p_ of only 90 mV. The decreased peak separation strongly indicates excellent catalytic activity of CS to the electro-oxidation of DA. The electrochemical process can be expressed by the following equation:

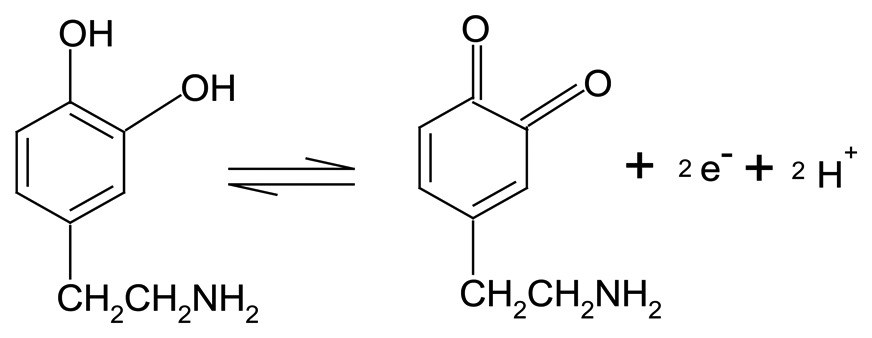


**Figure 5.**
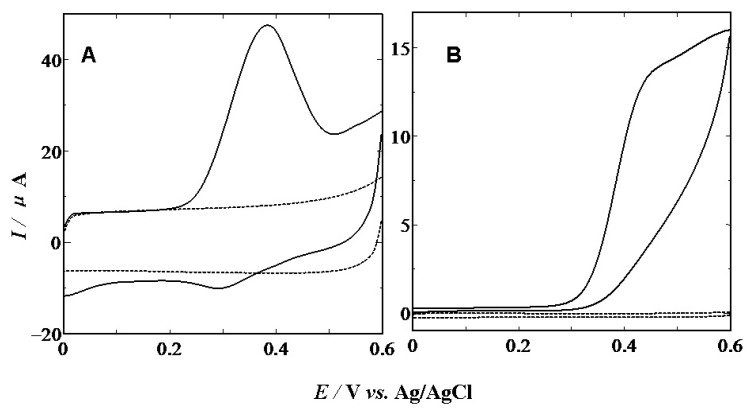
CVs obtained at CS/GCE (**A**), and bare GCE (**B**) electrode in the presence(solid line) or absence(dotted line) of 1.5 mM of 5-HT in the phosphate buffer solution (pH 7.0) with a scan rate of 100 m·Vs^−1^.

Figure 5 shows the CVs of 5-HT at CS modified (A) and bare (B) GCE, respectively. The voltammetric peak of 5-HT in the neutral pH 7.0 PBS appeared at about 0.46 V at the bare GC electrode (B) and the peak was rather broad, indicating a slow electron transfer kinetic. However, a sharp oxidation peak at 0.38 V and a small re-reduction peak at 0.3 V were obtained at CS/GC electrode (A). The 80 mV negative shift indicates a catalytic effect of the CS modified layer on the electro-oxidation of 5-HT. It must be pointed out that the appearance of the re-reduction peak of 5-HT oxidation indicates that reaction reversibility increases and the rate of following reactions at the CS/GCE reduces. The electrochemical process can be expressed as the following equation:

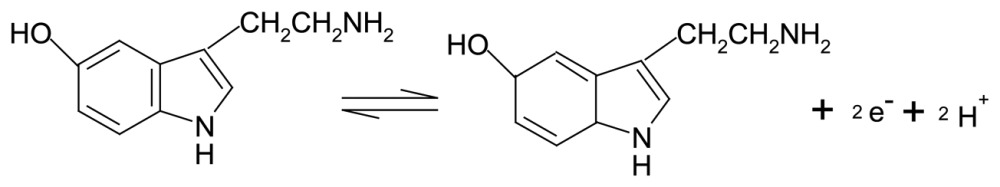


For the oxidation of DA and 5-HT, the anodic peak currents of DA and 5-HT are also both proportional to the scan rates and displayed a diffusion-controlled electrode reaction (data not shown).

Comparing with the bare electrode, the porous interfacial layer of the CS-modified electrode with a high specific surface area increases the conductive area, biomolecules can penetrate through the conductive porous channels onto the electrode more easily [23], leading to higher sensitivity and selectivity. On the other hand, edge plane of CS with its large number of edge plane sites facilitates and accelerates the electron-transfer rate between species and electrode [25] and therefore results in sensitive, well-defined and resolved signals for DA, 5-HT and AA, providing an electroanalytical method for the determination of DA, 5-HT and AA.

### 3.3. Simultaneous Determination of DA, 5-HT and AA at CS/GCE

**Figure 6.**
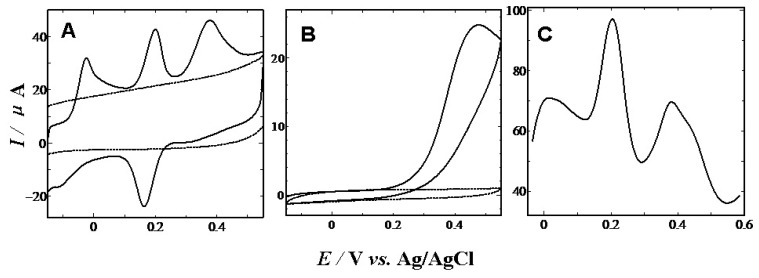
Cyclic voltammetry recordings of 2 mM AA, 0.5 mM DA and 0.5 mM HT at CS/GCE (**A**) and bare GCE (**B**) in 0.1 M phosphate buffer solution (pH 7.0) in the presence (solid line) and absence (dotted line) of AA, DA and HT with a scan rate of 100 mV·s^−1^; (**C**) Differential pulse voltammograms of the same mixture at CS/GCE.

Since DA, 5-HT and AA have similar oxidation potentials at most solid electrodes, separate determination of these species is a great problem due to their overlapping signals. In order to establish a sensitive and selective method for the quantification of DA, 5-HT and AA, the electrochemical oxidation of the mixture containing these three species at the CS modified GCE was studied. As shown in Figure 6B, the CV of the mixture solution containing DA, 5-HT and AA shows broad and overlapped anodic peaks at bare GCE, so the peak potentials for DA, 5-HT and AA are indistinguishable at a bare GCE and therefore, it is impossible to deduce any information from the broad and overlapped voltammetric peak. However, at the CS/GCE, the overlapped voltammetric peak is resolved into three well-defined anodic peaks at about −0.03, 0.20 and 0.38 V (A), corresponding to the oxidation of AA, DA and 5-HT, respectively, which are also observed in DPV mode (C). The separations of peaks were 230 mV, 180 mV and 410 mV between DA and AA, DA and 5-HT, and AA and 5-HT, respectively, which were large enough to determine DA, 5-HT and AA individually and simultaneously.

**Figure 7.**
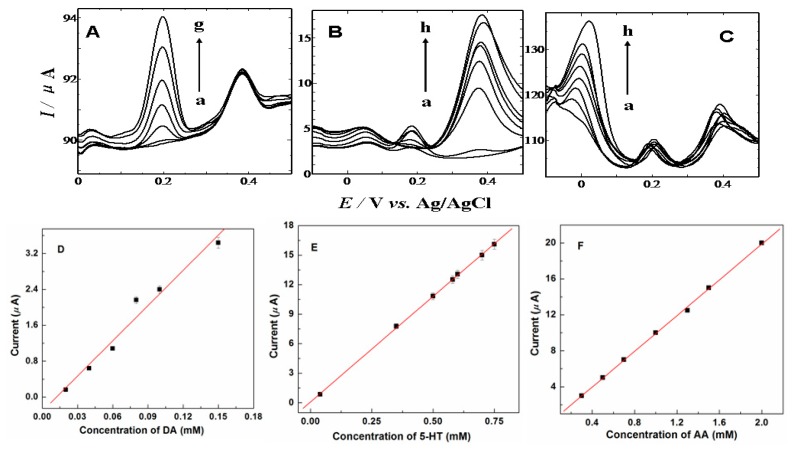
(**A**) DPVs of various concentrations of DA in 1 mM AA and 40 μM 5-HT solution (from a to g: 0, 0.02, 0.04, 0.06, 0.08, 0.1, 0.15 mM); (**B**) DPVs of various concentrations of HT in 1 mM AA and 12 μM DA solution (from a to h: 0, 0.04, 0.35, 0.5, 0.58, 0.6, 0.7, 0.75 mM); (**C**) DPVs of various concentrations of AA in 12 μM DA and 40 μM 5-HT solution (from a to h: 0, 0.3, 0.5, 0.7, 1.0, 1.3,1.5, 2.0 mM); (**D**)–(**F**) are the linear plots of currents against concentrations of DA, 5-HT, and AA, respectively.

The electro-oxidation processes of DA, AA and 5-HT in the mixture have also been investigated when the concentration of one species changed, whereas those of other two species are kept constant. DPV was employed to detect AA, DA and 5-HT because of its higher current sensitivity and better resolution than CV technique. Figure 7A gives the DPV recordings at various DA concentrations at the PVA modified GCE in the presence of AA and 5-HT. From Figure 7A, it can be seen that the peak current of DA increased with an increase in DA concentration when the concentrations of AA and 5-HT were kept constant. Similarly and obviously, as shown in Figure 7B,C, keeping the concentrations of other two compounds constant, the oxidation peak current of 5-HT and AA was positively proportional to its concentration, while those of other two compounds did not change. From our experimental results depicted above, it can be obtained that the electrochemical response peaks for DA, AA and 5-HT oxidation at the PVA modified GCE were clearly separated from each other when they co-exist in pH 7.0 PBS. It is therefore possible to simultaneously determine DA, AA and 5-HT in samples at a CS modified GCE. Under the optimum conditions, using the DPV mode, the catalytic current peak was linearly related to DA, AA and 5-HT concentration. The analytical parameters for the simultaneous determination of DA, 5-HT and AA are listed in Table 1.

### 3.4. Interferences, Stability and Repeatability

To evaluate the selectivity, we investigated the current response of CS/GCE to the ternary mixture containing 40 μM DA, 40 μM 5-HT, and 1 mM AA in the presence of different interfering substances. The effects of main relevant metal ions such as K^+^, Na^+^, Mg^2+^, Zn^2+^, Fe^2+^, Fe^3+^, Cl^−^, NO_3_^−^, SO_4_^2−^ on the current of the modified electrode for the mixture were studied and the results showed that the 1,000-fold excesses of Na^+^, K^+^, Mg^2+^, Zn^2+^, Cl^−^, NO_3_^−^, PO_4_^3−^, SO_4_^2−^, AC^−^, 700-fold excesses of Fe^2+^, 400-fold excesses of Fe^3+^, 10-fold excess of DOPAC, 5-fold excess of uric acid and 200-fold excess of oxalate, and 500-fold excess of glucose, L-cysteine, glutathione, folic acid, levodopa induced less than ±5% interference with the detection of DA, 5-HT, and AA. These results suggest that the CS/GC electrode possessed a good selective electrochemical response toward DA, 5-HT, and AA, and also indicated that CS/GC electrode could be used for the determination of DA, 5-HT, and AA in real samples.

The storage stability of CS/GCE was investigated by monitoring its current response to the ternary mixture containing 40 μM DA, 40 μM 5-HT, and 1 mM AA. The response of the electrode lost approximately 6.3%, 5.2%, and 4.8% of its original response for DA, 5-HT, and AA, respectively, after the storage of two weeks. The operational stability of the proposed electrode was also carried out by continuous assays by DPV of the ternary mixture containing 40 μM DA, 40 μM 5-HT, and 1 mM AA using one same CS/GCE in 2 h. A current decrease of *ca.* 5.3%, 6.2%, and 5.4% for DA, 5-HT, and AA was observed, respectively. The repeatability of the CS/GCE was also evaluated by measurements the same ternary mixture as above and the relative standard deviation (R.S.D.) for seven repeated measurements was found to be *ca.* 7.1%, 6.2%, and 6.4% for DA, 5-HT, and AA, respectively, suggesting that that CS/GCE is not subject to surface fouling by the oxidation products and is reproducible. Obviously, the proposed electrode shows a high stability and good reproducibility and anti-interference ability. Since the electrode fabrication is very easy and low cost, the present CS modified electrode seems to be of great utility for making electrochemical sensor for the detection of these three neurotransmitters.

### 3.5. Sample Analysis

The CS/GCE was applied to the amperometric assay of DA in dopamine hydrochloride injection (labeled as 10 mg·mL^−1^), AA in vitamin C injection (labeled as 200 mg·mL^−1^), and DA, 5-HT, AA in synthesized samples, respectively, by standard addition method. The injection solutions were diluted with PBS by 1,000 times and a ternary mixture containing 500 μM AA, 100 μM DA, and 100 μM 5-HT was used as synthesized samples. The results of the above assaying are listed in Table 2. The recoveries for the determinations vary from 96.5% to 105.5%, suggesting the proposed CS/GCE can be used to reliably determine DA, 5-HT and AA in real samples.

## 4. Conclusions

A novel carbon spheres-modified glassy carbon electrode was fabricated. The modified electrode shows good electrocatalytic activity for the electro-oxidation of DA, 5-HT and AA. Moreover, a better separation of oxidation peaks of DA, 5-HT and AA can be achieved, indicating that the carbon spheres-modified GCE facilitates the simultaneous determination of DA, 5-HT and AA with good stability, sensitivity and selectivity. The proposed method can be applied to the determination of DA, 5-HT and AA in real samples with satisfactory results.

## Figures and Tables

**Table 1.** Analytical parameters for the determination of DA, 5-HT, and AA.

**Analyte**	**Linear Range (mM)**	**Linear Regression Equation (*ΔI***: **μA; *C*: mM)**	**Correlation Coefficient (*r*)**	**Detection Limit (μM)**
DA	0.02–0.15	*ΔI* = −0.32 + 26.18 *C*	0.985	0.2
5-HT	0.04–0.75	*ΔI* = 0.09 + 21.44 *C*	0.999	0.7
AA	0.3–2.0	*ΔI* = −0.01 + 9.94 *C*	0.999	0.6

**Table 2.** Results of the determination of DA, 5-HT, and AA in real samples.

**Sample**	**Labeled** **(mg·mL^−1^)**	**Added** **(mg·mL^−1^)**	**Found** **(mg·mL^−1^)**	**R.S.D** **(%)**	**Recovery** **(%)**
vitamin C injection	200	0	194.2	1.9	-
		20	221.1	2.3	105.5
dopamine hydrochloride injection	10	0	10.1	3.1	-
		5	15.2	4.2	104.0
	labeled (μM)	added (μM)	found (μM)	R.S.D (%)	recovery (%)

synthesized sample	AA: 500	0	DA: 98.1	3.8	-
	DA: 100	DA: 20	119.3	4.7	96.5
	5-HT: 100				
	AA: 500	0	5-HT: 100.5	4.1	-
	DA: 100	5-HT: 20	120.3	3.9	101.5
	5-HT: 100				
	AA: 500	0	AA: 497.9	3.4	-
	DA: 100	AA:20	519.7	4.9	98.5
	5-HT: 100				
